# Evaluation of heating and liming treatments in sand samples artificially contaminated with *Ancylostoma* spp. eggs

**DOI:** 10.1590/S1984-29612024032

**Published:** 2024-06-17

**Authors:** Isabella Braghin Ferreira, Isabele Santos Garcia, Maria Linda Ferreira Lima, Rodrigo Costa da Silva, Vamilton Alvares Santarém

**Affiliations:** 1 Laboratório de Parasitologia Veterinária, Hospital Universitário Veterinário, Universidade do Oeste Paulista – UNOESTE, Presidente Prudente, SP, Brasil; 2 Faculdade de Pós-graduação em Zootecnia, Universidade do Oeste Paulista – UNOESTE, Presidente Prudente, SP, Brasil

**Keywords:** Soil contamination, geohelminth, cutaneous larva migrans, parasitic control, Contaminação do solo, geohelminto, larva migrans cutânea, controle parasitário

## Abstract

*Ancylostoma* spp. are found worldwide. Infected dog and cat feces can contaminate soil in public places. Despite prophylactic measures being available, studies on direct remediation of *Ancylostoma-*contaminated soils are scarce. This study aimed to determine the impact of heat treatment and liming on the viability of *Ancylostoma* spp. eggs in artificially contaminated sandy soil. Sterilized sand samples were contaminated with *Ancylostoma* spp. eggs extracted from infected dogs’ feces. Samples were heated (trial I) to 70 °C or 80 °C, then sieved after 24 hours (212, 90, 38, and 25 µm). Larval cultures were assessed for larval development following heat treatment. Five quicklime concentrations (trial II; 50, 30, 20, 10 and 5%) were used to treat sand. The effect of liming on larval cultures was assessed by measuring embryonic development. Filariform larvae were exposed to 20% quicklime (25 °C and 37 °C, 20 min). Heat treatment destroys *Ancylostoma* spp. eggs and prevents *in vitro* larval development. Liming at 50, 30, and 20% concentrations made embryonic development impossible. However, filariform larvae treated with 20% lime solution retained their motility. Heating at 70 °C and liming at 20% were sufficient to make *Ancylostoma* spp. egg embryogenesis impossible in experimentally contaminated sand samples.

## Resumo

*Ancylostoma* spp. são nematódeos que infectam cães e gatos e podem contaminar locais públicos. Apesar da existência de medidas profiláticas, estudos sobre tratamento do solo são escassos. No presente estudo, foi avaliado o efeito do tratamento térmico e caleação na viabilidade de ovos de *Ancylostoma* spp., em solo arenoso estéril. Amostras de solo foram contaminadas com ovos de *Ancylostoma* spp. obtidos de fezes de cães naturalmente infectados, aquecidas a 70°C ou 80°C, e filtradas em tamises metálicas (212, 90, 38 e 25 µm) após 24 horas (Teste I). Cultivos de larvas foram realizadas para a avaliação do desenvolvimento larval. Na caleação (Teste II), cinco concentrações (50, 30, 20, 10 e 5%) de cal virgem foram utilizadas. O efeito da caleação foi avaliado com observação do desenvolvimento larval nos cultivos. Ainda, larvas foram expostas (20 minutos) ao leite de cal (20% a 25°C e 37°C). O tratamento térmico foi capaz de degenerar ovos de *Ancylostoma* spp. e impedir o desenvolvimento larval, enquanto a caleação (50, 30, e 20%) impossibilitou o desenvolvimento larval. Entretanto, as larvas filariformes, expostas ao leite de cal (20%) mantiveram motilidade. O aquecimento (70°C) e caleação (20%) impediram o embrionamento de ovos de *Ancylostoma* spp. em solo experimentalmente contaminado.

## Introduction

*Ancylostoma* spp. are geohelminths of zoonotic importance that infect domestic carnivorous hosts such as dogs and cats worldwide (Bourgoin et al., 2022; Idrissi et al., 2022; Ola-Fadunsin et al., 2023).

Humans can potentially become infected through active penetration of the larva (L3) into the skin, which results in cutaneous larva migrans (CLM) (Bojar & Kłapeć, 2018). The penetration and migration of larvae through the skin causes serpiginous rash lesions with erythema and severe itching (Howard & Gibbs, 2019; Maxfield & Crane, 2024; Neupane et al., 2022). Lesions tend to be restricted to the skin and may occur on the feet (Harrison et al., 2022; Neupane et al., 2022), buttocks (Ansari et al., 2018; Magalhães & Nico, 2018), legs (Thadchanamoorthy & Dayasiri, 2021), arms (Sönmezer et al., 2017), back (Del Giudice et al., 2019), hands and abdomen (Eksomtramage & Aiempanakit, 2018).

CLM is most common in tropical and subtropical countries, although there has been an increase in cases in non-tropical regions as a result of global warming (Can & Yürekli, 2022). Autochthonous cases have been documented in European countries, including the United Kingdom (Howard & Gibbs, 2019), France (Del Giudice et al., 2019), and Germany (Kienast et al., 2007). This zoonosis is considered a traveler’s disease (Villagrasa-Boli et al., 2022; Ju et al., 2022), affecting tourists who have visited tropical countries and come into contact with soil (Smilga & Bujold, 2018; Gao & Liu, 2019). A retrospective analysis of diseases acquired in tropical countries found that dermatologic syndromes were prominent (40%), with CLM being the most common among travelers (27.0%) (Wilson et al., 2014).

Children are particularly susceptible to infection. Other risk factors for CLM are: being male, barefoot walking, and poverty (Reichert et al., 2018). CLM has recently been identified as a zoonosis associated with occupational risk in soil workers (Pulido-Pérez et al., 2019; Krzywanski et al., 2021; Stufano et al., 2022).

Free-roaming dogs and cats in public places may increase the chances of environmental contamination through helminth egg shedding via feces on soil (Dantas-Torres & Otranto, 2014; Traversa et al., 2014). A study in San Francisco de Campeche, Mexico, found that soil in public parks was contaminated with parasite eggs shed in dog feces (100%), including *A. caninum* (Cortez-Aguirre et al., 2018). *Ancylostoma* spp. are common in stray/free-roaming dogs and cats (Fu et al., 2019; Lyons et al., 2022), and these animals contribute to the contamination of public areas (Cortez-Aguirre et al., 2018). Several studies have reported soil contamination with *Ancylostoma* spp. in public places, universities, schools, clubs and beaches (Gallina et al., 2011; Rocha et al., 2011; Marques et al., 2012; Ribeiro et al., 2013; Sprenger et al., 2014; Leon et al., 2020; Silva et al., 2020; Lee et al., 2021; Mello et al., 2022; Nath et al., 2023).

CLM can be controlled by adopting prophylactic measures to reduce soil contamination from *Ancylostoma* spp. in dog and cat feces. Among these prophylactic practices, it is crucial to emphasize the eradication of feces in public places, the use of fencing, and responsible pet ownership through regular anthelmintic treatment (Traversa et al., 2014; Sprenger et al., 2014; Ola-Fadunsin et al., 2023). Effective control measures and population health education are needed when people’s knowledge of zoonotic parasites is limited (Idrissi et al., 2022).

Some disinfectants including ethanol (70% and 95%), 10% formalin, 10% povidone-iodine, 5% potassium dichromate, zinc sulfate-polyvinyl alcohol, and -20°C freezing have shown to be effective to inactivate *Ancylostoma* spp. eggs in the context of routine laboratory practices (Kines et al., 2021). For sludge samples, varying temperatures and quicklime concentrations have been used to inactivate helminth eggs (Capizzi-Banas et al., 2004; Maya et al., 2010). However, despite the significance of the CLM for public health, few studies are focusing on techniques for the direct treatment of contaminated soil. Therefore, the goal of the current study was to evaluate the efficiency of heating and liming treatments on sand samples that had been artificially contaminated with *Ancylostoma* spp. eggs.

## Material and Methods

### Sandy soil collection and sterilization

Sandy soil samples were obtained from a sandy field. The sand was dried at 80°C for 2 hours, and then sieved through a 250 µm mesh sieve using a Produtest^®^ shaker (LabX, Midland, Ontario, Canada) to remove large stones and grains and create homogenous soil. The sandy soil was then sterilized at 127 °C for 15 min using a Fabbe^®^ vertical sterilizer (Priorclave Ltd., London, UK) to eliminate any parasitic structure that could interfere with analysis.

### Obtaining *Ancylostoma* spp. eggs

*Ancylostoma* spp. eggs for sandy soil contamination were collected from the feces of naturally infected dogs that visited the Veterinary Hospital (Unoeste) on a regular basis. This study only included samples that demonstrated mono-parasitism caused by *Ancylostoma* eggs.

The Willis-Mollay technique was used to collect *Ancylostoma* spp. eggs (Hoffmann, 1987). Following flotation, the material that adhered to the slide was transferred to a centrifuge tube and centrifuged three times (873 *g*, 5 min) to concentrate the eggs. The supernatant was discharged after each centrifugation and purified water (OS10LXE model; Gehaka, São Paulo, Brazil) was added up to 10 mL q.s.

Under a 40x magnification microscope, aliquots of 100 (±5) *Ancylostoma* spp. eggs were isolated and stored in Eppendorf tubes with purified water (500 µL) until used for sandy soil contamination.

### Sand samples contamination

Sand samples were each weighed (10 g) in a 55 × 35 mm aluminum capsule (Solotest^®^, Bela Vista, São Paulo, Brazil) and contaminated with 100 (±5) *Ancylostoma* spp. eggs using a pipette (250 µL). Eppendorf tubes were washed with another 250 µL purified water to prevent egg adhesion to the tube and pipette tips. Following contamination, the samples were homogenized and used in the experimental treatments.

### Sand samples treatments

#### Trial I - Heating

Six contaminated sand samples were examined at both 70 °C and 80 °C. Two contaminated samples and one uncontaminated sample were used as positive and negative controls, respectively. The controls were not subjected to heat or liming.

A Bunsen burner was used for the heating treatment. Aluminum capsules containing contaminated sand were placed on a tripod with wire gauze and samples were heated to 70 or 80 ºC. The soil temperature was monitored using a skewer digital thermometer (SH-113J, Prolab, São Paulo, Brazil) positioned on the sample’s central surface and an infrared thermometer with ± 2 °C precision (MultiTemp°C, Porto Alegre, RS, Brazil). After heating, the samples were rehydrated with purified water (1.0 mL) and stored in an incubator (Fitotron^®^ SGC 120; Weiss Technik, Grand Rapids, MI, USA) for subsequent analysis. All samples were humidified with purified water (1.0 mL) five times per week to maintain soil moisture.

*Ancylostoma* spp. eggs were retrieved by sieving. Samples were rinsed with Tween 80 (75 mL) and washed in running water through metal mesh sieves (212, 90, 38, and 25 µm). Although *Ancylostoma* range from 51.78 ± 2.31µm by 36.37 ± 1.35µm in length and width, respectively (Lucio-Forster et al., 2012), in this study the washing process was previously tested, and it was observed that the eggs were retained in the 25 µm sieve. In this study, washing process was previously tested and it was observed that the eggs were retained in the 25 µm sieve. The pellet was examined under a microscope with magnifications of 10x and 40x. Eggs were considered viable when larvae were present inside but were non-viable when blastomere degeneration and/or wall disruption were seen. The differentiation between rhabditoid and filaroid larvae of *Ancylostoma* spp. was performed using the morphological criteria described by Guimarães et al. (2005).

Larvae were cultured using a modified Roberts and O’Sullivan technique (Hoffmann, 1987) to evaluate larval development following heating. For each temperature tested, larval culture was performed on six test samples (contaminated and treated), two positive controls (contaminated and untreated), and one negative control (no contamination, no treatment). The sand samples were placed in glass containers along with sterile sawdust (5.0 g) and dog feces (2.0 g) free of parasite structures. Cultures were homogenized, humidified with purified water (5.0 mL), covered with gauze, and stored in an incubator (Fitotron^®^; Weiss Technik) for 7 days at a temperature of 27±2 ºC. Cultures were humidified (2.0 mL) every 48 h.

The larvae were retrieved after 7 days of culture, which is sufficient time for *Ancylostoma* spp. larvae to develop into the infective form (Soulsby, 1982). Warm, purified water (39 °C) was added to each container until a meniscus formed. The cultures were placed in Petri dishes and incubated at ambient temperature (25 °C) for 3 h. The remaining water in the Petri dishes was then collected, transferred to conical tubes, and refrigerated at 7 °C for 3 hours to sediment the larvae. Following this process, the supernatant was removed, and the sediment was examined under a microscope (magnifications 4x and 10x).

#### Trial II – Liming

Five quicklime concentrations (50, 30, 20, 10, and 5%) were used in proportion to the amount of sand in the sample (10 g). For each quicklime concentration, six test samples (contaminated and quicklime-treated), two positive controls (contaminated and untreated), and one negative control (not contaminated and untreated) were evaluated. The liming treatment was performed by adding quicklime to the test samples. Larval cultures were prepared as described in the heating treatment section.

In addition, filariform larvae of *Ancylostoma* spp. collected from positive-controls samples were treated with a 20% lime solution to assess the effect of lime on the larvae. The larvae were transferred to 15 mL graduated tubes, centrifuged at 287 × *g* for 2 min, and the supernatant was discarded. A quicklime solution (20%; 1.0 mL; w/v) was prepared in purified water and added to the larvae. One tube was kept at ambient temperature (25 °C) and another in a water bath (37 °C) for 20 min. Each test was conducted using a control tube containing *Ancylostoma* spp. larvae in purified water (1 mL). After 20 min, the larvae were analyzed using a microscope (magnifications of 4x and 10x) to determine their motility.

### Data analysis

All data were entered into an Excel spreadsheet and examined for normalcy using the Kolmogorov-Smirnov test. Because the means followed a normal distribution, a non-paired t-test was used to compare them. All analyses were conducted using a significance level of 5%.

## Results

### Heating

**Figure 1:**
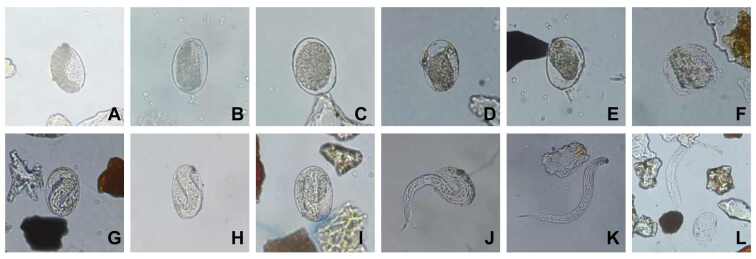
*Ancylostoma* spp. eggs and larvae recovered from samples 24 hours after thermal cleaning at 70 °C and 80 °C. Degenerated eggs retrieved from samples heated at 70 °C (A, B, C) and 80 °C (D, E, F) presented content compression, no cellular division (A, B, C, D, E, F), and eggshell deformation (E, F). Positive-control samples presented eggs containing larvae (G, H, I, J) and rhabditiform larvae (K, L). Magnification: 40x.

The samples took an average of 103 and 109 seconds to reach 70 °C and 80 °C, respectively. The percentages of eggs retrieved after 24 h of heating are shown in Table 1. The number of eggs retrieved after 24 h of heat treatment at 80 °C was significantly lower than that at 70 °C (P < 0.0001). All retrieved eggs, regardless of the temperature, were classified as deteriorated after heating (Figure 1). Eggs treated at 70 °C showed content compression and no cellular division. Samples heated at 80 °C showed deformed egg walls. The mean percentage of parasitic structures recovered in positive-control samples was 37.5% and 32.4% for testing at 70 ºC and at 80 °C, respectively. All eggs in these samples showed embryogenesis and most had larva (70 ºC = 74.1%; 80 ºC = 95.2%). Some rhabditiform larvae were also observed (70 °C = 25.9%; 80 ºC= 4.8%) (Figure 1).

**Table 1:** Mean percentage (± standard deviation) of *Ancylostoma* spp. eggs recovered from sand samples 24 hours after heat treatments at 70 °C or 80 °C.

Test	70 ºC (% ± sd)	80 ºC (% ± sd)
I	34.6	14.3
II	44.2	10.1
III	32.3	6.1
IV	33.0	12.6
V	37.1	6.8
VI	40.0	18.8
**Mean percentage (%)**	**36.87^A^ ± 4.6**	**11.45^B^ ± 4.8**

Means followed by different letters on the same line represent significant difference (p-value < 0.0001).

After seven days, no larvae were found in soil samples that were cultured and treated with heat, regardless of the temperature used. Positive-control samples showed a mean development of filariform larvae of 16.9% at 70 °C and 16.8% at 80 °C. All the larvae exhibited motility. The negative control samples did not exhibit any parasitic structures after heat treatment.

### Liming

The results obtained after liming are summarized in Table 2. Larval development was not observed in samples treated with 50%, 30%, or 20% quicklime. However, one filariform larva was seen in one sample from the 10% quicklime group. In addition, larval cultures treated with 5% quicklime had at least one filariform larva per sample. The mean percentage of filariform larval recovery in positive-control samples was 12.3% across all tests. All *Ancylostoma* spp. filariform larvae observed in this study were motile. The negative control samples did not exhibit any parasitic structures after liming.

**Table 2:** Mean percentage (%) of filariform larvae development in larvae culture inoculated with 100 *Ancylostoma* spp. eggs, seven days after liming (test samples) and without liming (positive control, PC).

Test		Quicklime concentrations (%)				
		50%	30%	20%	10%	5%
Test-samples	I	0	0	0	0	3
	II	0	0	0	1	2
	III	0	0	0	0	1
	IV	0	0	0	0	1
	V	0	0	0	0	2
	VI	0	0	0	0	1
**Mean percentage (%)**		**0**	**0**	**0**	**0.16**	**1.65**
PC	I	6.6	19.4	6.6	15.2	14
	II	15.4	6.2	19	12.4	8
**Mean percentage (%)**		**11**	**12.8**	**12.8**	**13.8**	**11**

PC: positive control.

Filariform larvae exposed to a 20% lime solution at different temperatures (25ºC and 37 ºC) retained their motility, demonstrating that the lime did not interfere with larvae motility.

## Discussion

To the best of our knowledge, this is the first study to evaluate treatment methods for soils contaminated with hookworm eggs. Our goal was to standardize techniques for heating (70 ºC and 80 ºC) and liming (50, 30, 20, 10, and 5%) treatments of sand that was experimentally contaminated with *Ancylostoma* spp. eggs.

Some studies have examined the efficacy of temperature and pH on the viability of helminth eggs, with most focusing on sludge treatment. Temperatures above 70 °C for 120 min effectively inactivated all helminth egg genera (Maya et al., 2010). Furthermore, Naidoo et al. (2018) found that *Ascaris suum* eggs were inactivated when exposed to an 80 °C water bath for four to five seconds, however at 75 °C and 70 °C more time is needed to achieve the same level of inactivation. Harroff et al. (2019) indicated that temperatures below 45 ºC could also inactivate *A. suum* eggs. The heating periods in this investigation were 103 and 109 seconds until reaching 70 ºC and 80 °C, respectively. However, the heating method differed from previous studies. We simulated a heating method that might be used in the environment while maintaining temperature control. We discovered that heating at 80 °C resulted in fewer eggs being recovered than heating at 70 °C, despite the fact that both temperatures interfered with egg viability. Heat treatment of *Ancylostoma* spp. eggs resulted in degenerative characteristics such as content compression, eggshell deformities, and the absence of embryogenesis. Because hookworm eggs are less resistant than *Ascaris* spp. eggs, studies focusing on temperature threshold may provide information on the minimum temperature required to inactivate *Ancylostoma* spp. eggs.

Regarding larval culture, we observed no larval development after seven days of heat treatment at either temperature. According to Soulsby (1982), the immature and infective *Ancylostoma* larvae are susceptible to desiccation. Therefore, periodic soil humidification allowed us to exclude the sample desiccation hypothesis. Thus, both temperatures may have stopped the development of *Ancylostoma* spp. larvae.

Liming has also been used for sewage sludge treatment (Capizzi-Banas et al., 2004; Herbst, 2000). Rossmann et al. (2014) observed that adding slaked lime at a concentration adequate to provide a pH of >12 reduced the viability of helminth eggs. We discovered that quicklime concentrations of 50, 30, and 20% inhibited *Ancylostoma* spp. larvae development, suggesting that liming may have affected egg viability. Ribeiro et al. (2017) investigated the effect of lime on *Strongyloides* spp. eggs and concluded that 10% quicklime (w/v) had a high ability to inactivate eggs in stalls of pacas (*Cuniculus paca*) raised in captivity, due the alkalinization of soil. In contrast, 10 and 5% quicklime concentrations were insufficient to impede *Ancylostoma* spp. egg development in this study.

Quicklime can be used to sanitize sewage sludge by destroying sludge pathogens as the temperature and pH increase (Capizzi-Banas et al., 2004; Herbst, 2000). Quicklime (calcium oxide) reacts exothermically with water to generate slacked lime (calcium hydroxide), which raises the pH (Atkins et al., 2013). The water used to humidify the samples probably caused a heat reaction that interfered with embryogenesis and eggshell membrane integrity, as seen in the heating treatment (trial I). As a limitation, the pH and temperature of samples used in the liming treatment were not evaluated. Further research into the efficiency of liming in soil samples could provide more information regarding temperature and pH values.

Although the results showed that liming at 20% was sufficient to render the eggs unviable, filariform larvae of *Ancylostoma* spp. treated with the same concentration of lime solution retained their motility. These results suggest that the action of lime is limited to *Ancylostoma* spp. eggs and may not affect larvae. One hypothesis explaining the resistance of filariform larvae (L3) to liming is the presence of a cuticle that protects the larva from chemical agents (Fortes, 2004).

The present study demonstrates the efficacy of the heating and liming treatment to inactivate *Ancylostoma* spp. eggs in soil under laboratory conditions. Therefore, further studies are necessary to evaluate the feasibility of *Ancylostoma* spp. eggs inactivation by heating or liming methods on large scale for application in public places.

## Conclusion

The heat treatment at 70°C and liming at 20% inhibited *Ancylostoma* spp. embryogenesis in experimentally contaminated sand samples. Our findings can serve as a foundation for future large-scale studies aimed at developing methods for soil treatment in public spaces contaminated with *Ancylostoma* spp., thereby benefiting public health.
